# Tris(5,6-dimethyl-1,10-phenanthroline-κ^2^ *N*,*N*′)iron(II) bis­(tricyano­methanide)

**DOI:** 10.1107/S1600536812046880

**Published:** 2012-11-24

**Authors:** Lucia Váhovská, Ivan Potočňák

**Affiliations:** a Institute of Chemistry, Faculty of Science, P.J. Šafárik University, Moyzesova 11, SK-041 54 Košice, Slovakia

## Abstract

The title compound, [Fe(C_14_H_12_N_2_)_3_](C_4_N_3_)_2_, consists of one [Fe(*dimephen*)_3_]^2+^ complex cation (*dimephen* = 5,6-dimethyl-1,10-phenanthroline) and two uncoordinating tcm anions (tcm = tricyano­methanide). In the complex cation, the Fe^II^ atom is coordinated by six N atoms from three chelating *dimephen* ligands at an average Fe—N distance of 1.963 (4) Å giving a distorted octa­hedral geometry. The crystal structure is stabilized by weak C—H⋯N hydrogen bonds and C N⋯π inter­actions between planar [maximum deviations of 0.024 (3) and 0.015 (3) Å] tcm anions and pyridine rings of *dimephen* [N2⋯centroid = 3.531 (3) and 3.726 (3) Å; C N⋯centroid = 96.4 (2) and 97.1 (2)°].

## Related literature

[Fe(*phen*)_2_(NCS)_2_] (*phen* = 1,10-phenathroline) and [Fe(*bpy*)_2_(NCS)_2_] (*bpy* = 2,2-bipyridine) are the first known and most extensively studied compounds of iron(II) exhibiting a high spin ←→ low spin transition, see: König & Watson (1970 ▶); Müller *et al.* (1982 ▶). For [Fe(*phen*)_3_]^2+^complexes, see: Aparici Plaza *et al.* (2007 ▶); Odoko & Okabe (2004 ▶); Koh *et al.* (1994 ▶); Uçar *et al.* (2005 ▶); Li *et al.* (2008 ▶). For bond lengths and angles in *dimephen*, see: Toledano-Magaña *et al.* (2012) ▶ and in tcm ligands, see: Potočňák *et al.* (2002 ▶); Luo *et al.* (2009 ▶). For the structure, properties and bonding modes of the tcm anion, see: Golub *et al.* (1986 ▶); Kohout *et al.* (2000 ▶). For the crystal and mol­ecular structure of *phen*, see: Nishigaki *et al.* (1978 ▶). For similar Fe^II^ complexes, see: Váhovská & Potočňák (2012 ▶).
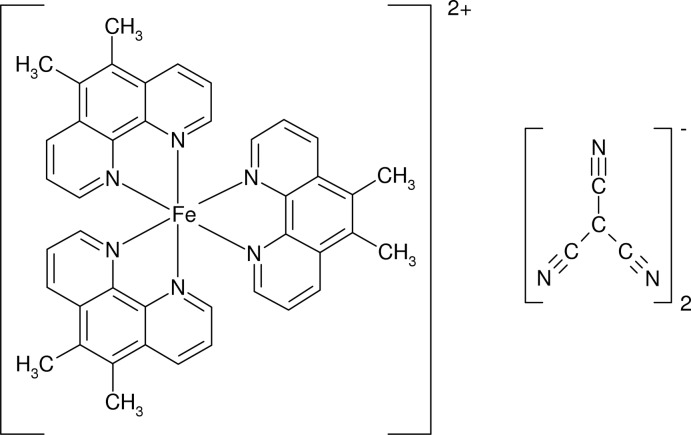


## Experimental

### 

#### Crystal data

[Fe(C_14_H_12_N_2_)_3_](C_4_N_3_)_2_
*M* *_r_* = 860.76
Triclinic, 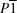

*a* = 9.3676 (3) Å
*b* = 12.7079 (9) Å
*c* = 18.1998 (9) Å
α = 75.458 (5)°
β = 89.623 (3)°
γ = 82.323 (4)°
*V* = 2077.52 (19) Å^3^
*Z* = 2
Mo *K*α radiation
μ = 0.42 mm^−1^
*T* = 183 K
0.66 × 0.25 × 0.03 mm

#### Data collection

Agilent Xcalibur (Sapphire2) diffractometer
Absorption correction: analytical [*CrysAlis PRO* (Agilent, 2012 ▶), based on expressions derived by Clark & Reid (1995 ▶)] *T*_min_ = 0.874, *T*_max_ = 0.986
15408 measured reflections
8167 independent reflections
6309 reflections with *I* > 2σ(*I*)
*R*_int_ = 0.025

#### Refinement

*R*[*F* ^2^ > 2σ(*F* ^2^)] = 0.050
*wR*(*F* ^2^) = 0.125
*S* = 1.07
8167 reflections
574 parameters
H-atom parameters constrained
Δρ_max_ = 0.92 e Å^−3^
Δρ_min_ = −0.40 e Å^−3^

### 

Data collection: *CrysAlis PRO* (Agilent, 2012 ▶); cell refinement: *CrysAlis PRO*; data reduction: *CrysAlis PRO*; program(s) used to solve structure: *SHELXS97* (Sheldrick, 2008 ▶); program(s) used to refine structure: *SHELXL97* (Sheldrick, 2008 ▶); molecular graphics: *DIAMOND* (Brandenburg, 2001 ▶); software used to prepare material for publication: *SHELXL97*.

## Supplementary Material

Crystal structure: contains datablock(s) I, global. DOI: 10.1107/S1600536812046880/bx2428sup1.cif

Structure factors: contains datablock(s) I. DOI: 10.1107/S1600536812046880/bx2428Isup2.hkl

Additional supplementary materials:  crystallographic information; 3D view; checkCIF report

## Figures and Tables

**Table 1:** Selected bond lengths (Å)

Fe1—N20	1.957 (2)
Fe1—N10	1.959 (2)
Fe1—N60	1.963 (2)
Fe1—N30	1.965 (2)
Fe1—N50	1.967 (2)
Fe1—N40	1.968 (2)

**Table 2:** Hydrogen-bond geometry (Å, °)

*D*—H⋯*A*	*D*—H	H⋯*A*	*D*⋯*A*	*D*—H⋯*A*
C52—H52⋯N30	0.95	2.59	3.070 (3)	112
C22—H22⋯N40	0.95	2.59	3.089 (4)	113
C32—H32⋯N8	0.95	2.43	3.266 (4)	146
C42—H42⋯N60	0.95	2.57	3.056 (3)	112
C22—H22⋯N6^i^	0.95	2.54	3.271 (4)	134
C12—H12⋯N2^ii^	0.95	2.55	3.352 (4)	142
C62—H62⋯N3^iii^	0.95	2.51	3.266 (4)	137
C44—H44⋯N7^iv^	0.95	2.59	3.312 (4)	133

Symmetry codes: (i) 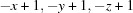; (ii) 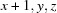; (iii) 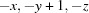; (iv) 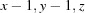.

**Fig. 1.**
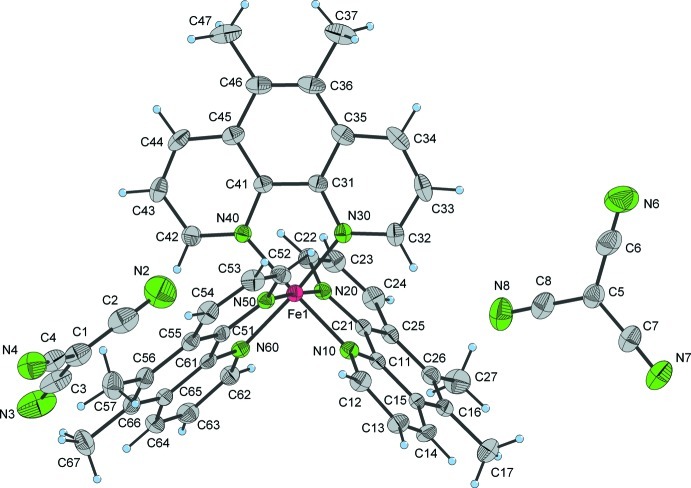
The structure of the title compound.

**Fig. 2.**
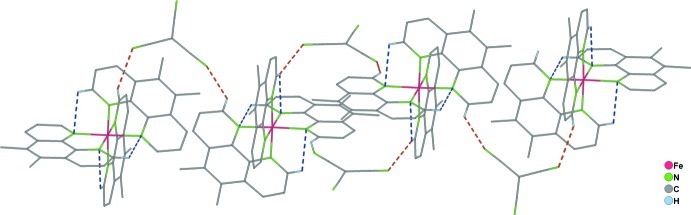
Intramolecular and intermolecular C—H···N hydrogen bonds (blue and red dashed lines, respectively) in the title compound. H-atoms not involved in hydrogen bonds are omitted because of clarity.

**Fig. 3.**
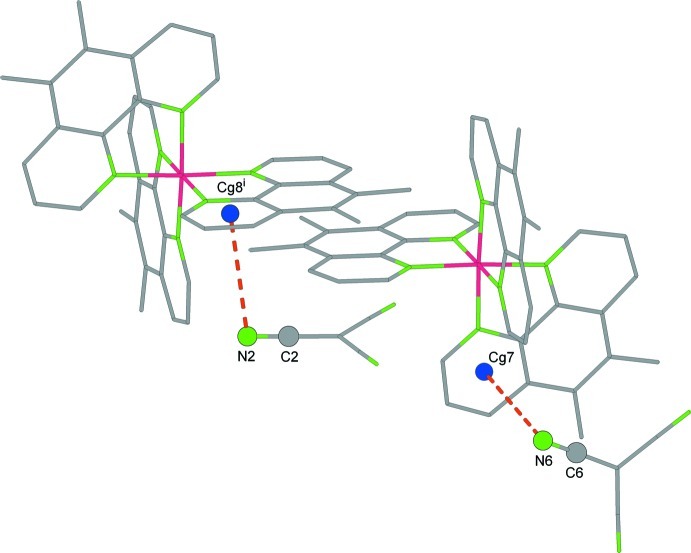
π-π interactions (dashed lines) between tcm and pyridine rings in the title compound (symmetry codes: (i) = x –1, y, z). H-atoms are omitted because of clarity.

## supplementary crystallographic information

### Comment

The iron(II) complexes [Fe(*phen*)_2_(NCS)_2_] and [Fe(*bpy*)_2_(NCS)_2_] (*bpy* = 2,2-bipyridine) belong to the first known and most extensively studied compounds of iron(II) exhibiting a high spin ←→ low spin transition (Müller *et al.*, 1982; König & Watson, 1970). By far the majority of known spin-transition compounds are octahedral Fe^II^ compounds of general formula [Fe(*L*)_4_(NCX)_2_] or [Fe(*L*)_2_(NCX)_2_] (*L* = monodentate or bidentate N-donor ligands, X = S, Se). In our research, which is aimed on preparation of new [Fe(*L*)_2_(Y)_2_] compounds (*L* = *bpy*, *phen* or their derivatives and Y = pseudohalide anions (dicyanamide, or tcm)) with possible spin crossover, we prepared crystals of the title compound with composition [Fe(*dimephen*)_3_](tcm)_2_ (I) (*dimephen* = 5,6-dimethyl-1,10-phenanthroline).

Structural analysis showed that crystal structure of the title compound is ionic and consists of one complex cation and two tcm counter-anions (Fig. 1).

In the complex cation the Fe^II^ ion is bonded to three bidentate *dimephen* ligands through their nitrogen atoms resulting in a distorted octahedral arrangement with the six Fe1–N distances ranging from 1.957 (2) to 1.968 (2) Å (Table 1). These values as well as the values of N–Fe1–N bite angles (82.88 (9), 82.69 (9), 82.44 (9)°) and opposite (*trans*) angles (173.92 (9), 176.49 (9), 176.56 (9)°) are comparable to the corresponding distances and angles in other complexes with [Fe(*phen*)_3_]^2+^ cations (Aparici Plaza *et al.*, 2007; Odoko & Okabe, 2004; Koh *et al.*, 1994; Uçar *et al.*, 2005; Li *et al.*, 2008). All N–Fe1–N bond angles in (I) deviate significantly from the ideal values of 90 or 180° because of the constrained geometry of the *dimephen* ring systems. The values of bond distances and angles within the rings of neutral ligands are similar to those found in the similar [Cu(*dimephen*)_3_](PF_6_)_2_.CH_3_CN complex, too (Toledano-Magaña *et al.*, 2012). The *dimephen* ligands in (I) are almost planar, the largest deviation of atom from the mean plane being 0.051 (3) Å for atom C63.

Both tcm anions are nearly planar, too (the largest deviations of atoms from the mean planes being 0.024 (3) for C1 atom and 0.015 (3) Å for C5 atom). The average C–C and C≡N bond lengths (1.404 (6) and 1.155 (3) Å, respectively), C–C–C (120.0 (3)°) and C–C≡N (179.0 (6)°) angles within the both anions are in good agreement with those found in other tricyanomethanide complexes (Potočňák *et al.*, 2002; Luo *et al.*, 2009).

The crystal packing in (I) is formed by weak C–H···N hydrogen bonds (Table 2) and C–N···*Cg*π-ring interactions. Weak hydrogen bonds occur between individual *dimephen* ligands and thus the structure of the cation is stabilized. Moreover, tcm anions interconnect two [Fe(*dimephen*)_3_]^2+^cations through hydrogen bonds and these interactions lead to infinite chain-like structure running along *z* axis (Fig. 2).

Except hydrogen bonds, the crystal structure is stabilized by π-ring interactions between nitrogen atoms from tcm anions and corresponding pyridine rings. The N2···*Cg*8^i^ (i = *x* – 1, *y*, *z*) and N6···*Cg*7 distances (3.531 (3) and 3.726 (3) Å, respectively, *Cg*8 and *Cg*7 are centroids of pyridine rings with N50 and N40 atoms, respectively), the distances of N2 and N6 atoms to the planes of the corresponding *dimephen* rings (3.505 and 3.677 Å, respectively) as well as the C2≡N2···*Cg*8^i^ and C6≡N6···*Cg*7 angles (96.4 (2) and 97.1 (2)°, respectively) are close to those found in similar Fe^II^ complexes (Váhovská & Potočňák, 2012). Parallel arrangement of tcm anions with *dimephen* molecules in (I) is shown in Fig. 3.

### Experimental

Single crystals of the title compound were obtained at the interfaces of layered systems, with the lower layer comprising an aqueous solution (5 ml) of iron(II) sulfate (0.1 mmol) and 5 ml of tcm (0.1 mmol) and the upper layer comprising a methanolic solution (3 ml) of *dimephen* (0.1 mmol). These layered systems were allowed to stand at room temperature. Red crystals suitable for X-ray analysis were obtained and filtered off in several days and dried on air.

### Refinement

Anisotropic displacement parameters were refined for all non-H atoms. The aromatic as well as methyl H atoms were placed in calculated positions and refined riding on their parent C atoms with C–H distances of 0.95 and 0.98 Å, respectively and *U*_iso_(H) = 1.2*U*_eq_(C) and 1.5*U*_eq_(C) for aromatic and methyl hydrogen atoms, respectively.

### Crystal data

**Table:**

[Fe(C_14_H_12_N_2_)_3_](C_4_N_3_)_2_	*Z* = 2
*M**_r_* = 860.76	*F*(000) = 892
Triclinic, *P*1	*D*_x_ = 1.376 Mg m^−^^3^
Hall symbol: -P 1	Mo *K*α radiation, λ = 0.71073 Å
*a* = 9.3676 (3) Å	Cell parameters from 5036 reflections
*b* = 12.7079 (9) Å	θ = 3.0–29.2°
*c* = 18.1998 (9) Å	µ = 0.42 mm^−^^1^
α = 75.458 (5)°	*T* = 183 K
β = 89.623 (3)°	Needle, dark red
γ = 82.323 (4)°	0.66 × 0.25 × 0.03 mm
*V* = 2077.52 (19) Å^3^	

### Data collection

**Table:**

Agilent Xcalibur (Sapphire2) diffractometer	8167 independent reflections
Radiation source: fine-focus sealed tube	6309 reflections with *I* > 2σ(*I*)
Graphite monochromator	*R*_int_ = 0.025
Detector resolution: 8.3438 pixels mm^-1^	θ_max_ = 26.0°, θ_min_ = 3.0°
ω scans	*h* = −11→11
Absorption correction: analytical [*CrysAlis PRO* (Agilent, 2012), based on expressions derived by Clark & Reid (1995)]	*k* = −15→10
*T*_min_ = 0.874, *T*_max_ = 0.986	*l* = −22→21
15408 measured reflections	

### Refinement

**Table:**

Refinement on *F*^2^	Primary atom site location: structure-invariant direct methods
Least-squares matrix: full	Secondary atom site location: difference Fourier map
*R*[*F*^2^ > 2σ(*F*^2^)] = 0.050	Hydrogen site location: inferred from neighbouring sites
*wR*(*F*^2^) = 0.125	H-atom parameters constrained
*S* = 1.07	*w* = 1/[σ^2^(*F*_o_^2^) + (0.0473*P*)^2^ + 1.1913*P*] where *P* = (*F*_o_^2^ + 2*F*_c_^2^)/3
8167 reflections	(Δ/σ)_max_ = 0.001
574 parameters	Δρ_max_ = 0.92 e Å^−^^3^
0 restraints	Δρ_min_ = −0.40 e Å^−^^3^

### Special details

**Table:**

Experimental. CrysAlis PRO (Agilent, 2012) Analytical numeric absorption correction using a multifaceted crystal model based on expressions derived by R.C. Clark & J.S. Reid. (Clark, R. C. & Reid, J. S. (1995). Acta Cryst. A51, 887-897)
Geometry. All e.s.d.'s (except the e.s.d. in the dihedral angle between two l.s. planes) are estimated using the full covariance matrix. The cell e.s.d.'s are taken into account individually in the estimation of e.s.d.'s in distances, angles and torsion angles; correlations between e.s.d.'s in cell parameters are only used when they are defined by crystal symmetry. An approximate (isotropic) treatment of cell e.s.d.'s is used for estimating e.s.d.'s involving l.s. planes.
Refinement. Refinement of *F*^2^ against ALL reflections. The weighted *R*-factor *wR* and goodness of fit *S* are based on *F*^2^, conventional *R*-factors *R* are based on *F*, with *F* set to zero for negative *F*^2^. The threshold expression of *F*^2^ > σ(*F*^2^) is used only for calculating *R*-factors(gt) *etc*. and is not relevant to the choice of reflections for refinement. *R*-factors based on *F*^2^ are statistically about twice as large as those based on *F*, and *R*- factors based on ALL data will be even larger.

### Fractional atomic coordinates and isotropic or equivalent isotropic displacement parameters (Å^2^)

**Table:**

	*x*	*y*	*z*	*U*_iso_*/*U*_eq_	
Fe1	0.52758 (4)	0.38724 (3)	0.27263 (2)	0.01786 (11)	
N10	0.6319 (2)	0.51196 (18)	0.23141 (12)	0.0199 (5)	
N20	0.3666 (2)	0.50317 (18)	0.27106 (12)	0.0196 (5)	
N30	0.5692 (2)	0.37913 (18)	0.37975 (12)	0.0208 (5)	
N40	0.4163 (2)	0.26722 (18)	0.31777 (12)	0.0189 (5)	
N50	0.6912 (2)	0.27509 (18)	0.26764 (12)	0.0196 (5)	
N60	0.4893 (2)	0.37825 (18)	0.16864 (12)	0.0191 (5)	
C11	0.5467 (3)	0.6115 (2)	0.22050 (14)	0.0178 (5)	
C12	0.7678 (3)	0.5128 (2)	0.20913 (15)	0.0250 (6)	
H12	0.8295	0.4451	0.2168	0.030*	
C13	0.8222 (3)	0.6097 (2)	0.17501 (16)	0.0279 (7)	
H13	0.9186	0.6070	0.1583	0.034*	
C14	0.7372 (3)	0.7085 (2)	0.16549 (16)	0.0273 (7)	
H14	0.7750	0.7746	0.1430	0.033*	
C15	0.5938 (3)	0.7123 (2)	0.18903 (14)	0.0204 (6)	
C16	0.4937 (3)	0.8123 (2)	0.18188 (15)	0.0235 (6)	
C17	0.5497 (3)	0.9199 (3)	0.14961 (18)	0.0365 (8)	
H17A	0.4687	0.9793	0.1376	0.055*	
H17B	0.6020	0.9165	0.1033	0.055*	
H17C	0.6148	0.9337	0.1870	0.055*	
C21	0.4017 (3)	0.6066 (2)	0.24352 (14)	0.0184 (6)	
C22	0.2318 (3)	0.4941 (2)	0.29416 (16)	0.0255 (6)	
H22	0.2046	0.4230	0.3132	0.031*	
C23	0.1304 (3)	0.5858 (3)	0.29106 (17)	0.0297 (7)	
H23	0.0362	0.5767	0.3089	0.036*	
C24	0.1652 (3)	0.6885 (3)	0.26263 (16)	0.0284 (7)	
H24	0.0952	0.7508	0.2603	0.034*	
C25	0.3061 (3)	0.7028 (2)	0.23650 (15)	0.0209 (6)	
C26	0.3543 (3)	0.8075 (2)	0.20441 (15)	0.0245 (6)	
C27	0.2448 (3)	0.9083 (3)	0.19628 (19)	0.0389 (8)	
H27A	0.2917	0.9740	0.1775	0.058*	
H27B	0.2035	0.9090	0.2458	0.058*	
H27C	0.1680	0.9073	0.1602	0.058*	
C31	0.5127 (3)	0.2963 (2)	0.42907 (14)	0.0201 (6)	
C32	0.6471 (3)	0.4402 (2)	0.40944 (16)	0.0257 (6)	
H32	0.6869	0.4988	0.3763	0.031*	
C33	0.6717 (3)	0.4202 (3)	0.48748 (17)	0.0326 (7)	
H33	0.7272	0.4652	0.5067	0.039*	
C34	0.6164 (3)	0.3365 (3)	0.53644 (17)	0.0320 (7)	
H34	0.6342	0.3228	0.5896	0.038*	
C35	0.5328 (3)	0.2702 (2)	0.50797 (15)	0.0266 (6)	
C36	0.4690 (3)	0.1787 (3)	0.55442 (16)	0.0300 (7)	
C37	0.4985 (3)	0.1533 (3)	0.63885 (17)	0.0434 (9)	
H37A	0.4570	0.0871	0.6641	0.065*	
H37B	0.4548	0.2151	0.6582	0.065*	
H37C	0.6027	0.1411	0.6491	0.065*	
C41	0.4288 (2)	0.2355 (2)	0.39481 (14)	0.0187 (6)	
C42	0.3382 (3)	0.2126 (2)	0.28329 (16)	0.0256 (6)	
H42	0.3264	0.2345	0.2296	0.031*	
C43	0.2731 (3)	0.1237 (2)	0.32412 (18)	0.0301 (7)	
H43	0.2195	0.0855	0.2978	0.036*	
C44	0.2859 (3)	0.0914 (2)	0.40121 (17)	0.0293 (7)	
H44	0.2412	0.0310	0.4285	0.035*	
C45	0.3654 (3)	0.1477 (2)	0.44031 (16)	0.0243 (6)	
C46	0.3868 (3)	0.1207 (2)	0.52172 (16)	0.0286 (7)	
C47	0.3141 (3)	0.0276 (3)	0.56809 (19)	0.0422 (9)	
H47A	0.3495	0.0093	0.6210	0.063*	
H47B	0.3361	−0.0368	0.5475	0.063*	
H47C	0.2097	0.0499	0.5659	0.063*	
C51	0.6892 (3)	0.2399 (2)	0.20271 (15)	0.0188 (6)	
C52	0.7946 (3)	0.2252 (2)	0.31962 (15)	0.0239 (6)	
H52	0.7994	0.2494	0.3648	0.029*	
C53	0.8950 (3)	0.1397 (2)	0.31027 (16)	0.0278 (7)	
H53	0.9671	0.1066	0.3486	0.033*	
C54	0.8910 (3)	0.1025 (2)	0.24603 (17)	0.0277 (7)	
H54	0.9586	0.0426	0.2403	0.033*	
C55	0.7863 (3)	0.1536 (2)	0.18851 (15)	0.0216 (6)	
C56	0.7733 (3)	0.1236 (2)	0.11727 (16)	0.0258 (6)	
C57	0.8814 (3)	0.0306 (3)	0.10493 (19)	0.0368 (8)	
H57A	0.8544	0.0109	0.0587	0.055*	
H57B	0.8824	−0.0332	0.1485	0.055*	
H57C	0.9774	0.0537	0.0996	0.055*	
C61	0.5799 (3)	0.2973 (2)	0.14812 (14)	0.0192 (6)	
C62	0.3875 (3)	0.4362 (2)	0.11791 (15)	0.0232 (6)	
H62	0.3234	0.4930	0.1307	0.028*	
C63	0.3724 (3)	0.4158 (2)	0.04676 (16)	0.0289 (7)	
H63	0.3001	0.4597	0.0117	0.035*	
C64	0.4609 (3)	0.3332 (2)	0.02716 (16)	0.0272 (6)	
H64	0.4491	0.3188	−0.0210	0.033*	
C65	0.5700 (3)	0.2695 (2)	0.07885 (15)	0.0214 (6)	
C66	0.6681 (3)	0.1789 (2)	0.06461 (16)	0.0252 (6)	
C67	0.6472 (3)	0.1516 (3)	−0.00986 (18)	0.0382 (8)	
H67A	0.7072	0.0823	−0.0100	0.057*	
H67B	0.6751	0.2103	−0.0513	0.057*	
H67C	0.5457	0.1444	−0.0169	0.057*	
C1	0.0574 (3)	0.2816 (3)	0.04263 (18)	0.0358 (8)	
C2	0.0526 (3)	0.3207 (3)	0.1087 (2)	0.0436 (9)	
C3	−0.0500 (3)	0.3222 (3)	−0.0146 (2)	0.0438 (9)	
C4	0.1662 (3)	0.1985 (3)	0.03535 (18)	0.0338 (7)	
C5	0.9633 (3)	0.7346 (3)	0.44900 (18)	0.0325 (7)	
C6	0.9720 (3)	0.6902 (3)	0.5285 (2)	0.0413 (8)	
C7	1.0418 (3)	0.8197 (3)	0.41576 (19)	0.0348 (7)	
C8	0.8731 (3)	0.6955 (3)	0.4037 (2)	0.0374 (8)	
N2	0.0493 (3)	0.3516 (3)	0.1633 (2)	0.0640 (10)	
N3	−0.1365 (3)	0.3538 (3)	−0.0629 (2)	0.0624 (10)	
N4	0.2554 (3)	0.1297 (2)	0.02865 (17)	0.0470 (7)	
N6	0.9798 (3)	0.6547 (3)	0.5933 (2)	0.0670 (10)	
N7	1.1049 (3)	0.8905 (2)	0.38654 (18)	0.0482 (8)	
N8	0.7995 (3)	0.6636 (3)	0.36574 (19)	0.0508 (8)	

### Atomic displacement parameters (Å^2^)

**Table:**

	*U*^11^	*U*^22^	*U*^33^	*U*^12^	*U*^13^	*U*^23^
Fe1	0.01556 (18)	0.0189 (2)	0.0196 (2)	−0.00280 (14)	0.00151 (14)	−0.00547 (16)
N10	0.0156 (10)	0.0235 (13)	0.0207 (12)	−0.0017 (9)	0.0029 (8)	−0.0062 (10)
N20	0.0175 (10)	0.0233 (13)	0.0196 (12)	−0.0040 (9)	0.0034 (9)	−0.0076 (10)
N30	0.0171 (10)	0.0223 (13)	0.0242 (12)	−0.0025 (9)	0.0005 (9)	−0.0084 (10)
N40	0.0183 (10)	0.0186 (12)	0.0201 (12)	−0.0026 (9)	0.0005 (9)	−0.0053 (10)
N50	0.0179 (11)	0.0215 (13)	0.0194 (12)	−0.0052 (9)	0.0005 (9)	−0.0037 (10)
N60	0.0160 (10)	0.0191 (12)	0.0212 (12)	−0.0028 (9)	0.0024 (8)	−0.0031 (10)
C11	0.0196 (13)	0.0199 (15)	0.0146 (13)	−0.0021 (11)	−0.0009 (10)	−0.0061 (11)
C12	0.0175 (13)	0.0281 (17)	0.0293 (16)	−0.0011 (11)	0.0047 (11)	−0.0085 (13)
C13	0.0206 (14)	0.0351 (18)	0.0301 (16)	−0.0108 (13)	0.0078 (11)	−0.0084 (14)
C14	0.0287 (15)	0.0288 (17)	0.0254 (15)	−0.0153 (13)	0.0050 (12)	−0.0029 (13)
C15	0.0255 (13)	0.0234 (15)	0.0139 (13)	−0.0084 (11)	−0.0018 (10)	−0.0048 (11)
C16	0.0327 (15)	0.0222 (16)	0.0160 (14)	−0.0067 (12)	−0.0026 (11)	−0.0039 (12)
C17	0.0437 (18)	0.0239 (17)	0.0416 (19)	−0.0100 (14)	0.0025 (14)	−0.0050 (15)
C21	0.0198 (13)	0.0200 (15)	0.0161 (13)	−0.0027 (11)	−0.0002 (10)	−0.0061 (11)
C22	0.0199 (13)	0.0304 (17)	0.0282 (15)	−0.0060 (12)	0.0048 (11)	−0.0098 (13)
C23	0.0180 (13)	0.0391 (19)	0.0336 (17)	−0.0033 (12)	0.0059 (12)	−0.0125 (15)
C24	0.0200 (13)	0.0308 (18)	0.0341 (17)	0.0050 (12)	0.0018 (12)	−0.0119 (14)
C25	0.0223 (13)	0.0220 (15)	0.0184 (14)	0.0012 (11)	−0.0027 (10)	−0.0075 (12)
C26	0.0306 (15)	0.0230 (16)	0.0192 (14)	−0.0007 (12)	−0.0018 (11)	−0.0056 (12)
C27	0.0429 (18)	0.0287 (19)	0.0399 (19)	0.0068 (14)	0.0013 (14)	−0.0050 (15)
C31	0.0177 (12)	0.0223 (15)	0.0195 (14)	0.0006 (11)	0.0032 (10)	−0.0055 (12)
C32	0.0221 (13)	0.0271 (16)	0.0314 (16)	−0.0032 (12)	−0.0009 (11)	−0.0140 (13)
C33	0.0304 (15)	0.0380 (19)	0.0353 (18)	−0.0009 (13)	−0.0075 (13)	−0.0220 (15)
C34	0.0325 (16)	0.043 (2)	0.0219 (15)	0.0045 (14)	−0.0036 (12)	−0.0161 (15)
C35	0.0224 (14)	0.0332 (18)	0.0221 (15)	0.0061 (12)	−0.0005 (11)	−0.0084 (13)
C36	0.0256 (14)	0.0362 (19)	0.0209 (15)	0.0083 (13)	0.0044 (11)	−0.0006 (13)
C37	0.0424 (18)	0.057 (2)	0.0242 (17)	0.0025 (16)	0.0032 (14)	−0.0024 (16)
C41	0.0139 (12)	0.0195 (15)	0.0215 (14)	0.0005 (10)	0.0032 (10)	−0.0042 (11)
C42	0.0232 (14)	0.0291 (17)	0.0254 (15)	−0.0065 (12)	−0.0005 (11)	−0.0071 (13)
C43	0.0246 (14)	0.0270 (17)	0.0424 (19)	−0.0093 (12)	−0.0004 (12)	−0.0123 (14)
C44	0.0245 (14)	0.0219 (16)	0.0405 (18)	−0.0087 (12)	0.0082 (12)	−0.0033 (14)
C45	0.0180 (13)	0.0234 (16)	0.0286 (15)	0.0001 (11)	0.0050 (11)	−0.0029 (13)
C46	0.0231 (14)	0.0311 (17)	0.0254 (16)	0.0039 (12)	0.0060 (11)	0.0002 (13)
C47	0.0385 (17)	0.041 (2)	0.0369 (19)	−0.0039 (15)	0.0125 (14)	0.0073 (16)
C51	0.0170 (12)	0.0196 (14)	0.0216 (14)	−0.0074 (10)	0.0051 (10)	−0.0062 (11)
C52	0.0221 (13)	0.0296 (17)	0.0197 (14)	−0.0034 (12)	−0.0018 (11)	−0.0054 (12)
C53	0.0190 (13)	0.0322 (18)	0.0287 (16)	0.0007 (12)	−0.0032 (11)	−0.0029 (14)
C54	0.0201 (13)	0.0227 (16)	0.0393 (18)	0.0016 (11)	0.0034 (12)	−0.0082 (14)
C55	0.0177 (12)	0.0201 (15)	0.0276 (15)	−0.0054 (11)	0.0044 (11)	−0.0053 (12)
C56	0.0227 (14)	0.0236 (16)	0.0350 (17)	−0.0060 (11)	0.0085 (12)	−0.0132 (13)
C57	0.0303 (16)	0.038 (2)	0.049 (2)	−0.0019 (14)	0.0074 (14)	−0.0248 (17)
C61	0.0202 (13)	0.0167 (14)	0.0211 (14)	−0.0073 (10)	0.0056 (10)	−0.0033 (11)
C62	0.0221 (13)	0.0220 (15)	0.0246 (15)	−0.0028 (11)	−0.0002 (11)	−0.0040 (12)
C63	0.0287 (15)	0.0292 (17)	0.0256 (16)	−0.0056 (13)	−0.0060 (12)	−0.0001 (13)
C64	0.0330 (15)	0.0312 (17)	0.0190 (14)	−0.0096 (13)	−0.0005 (11)	−0.0064 (13)
C65	0.0235 (13)	0.0227 (15)	0.0200 (14)	−0.0095 (11)	0.0028 (10)	−0.0059 (12)
C66	0.0263 (14)	0.0258 (16)	0.0279 (16)	−0.0109 (12)	0.0076 (12)	−0.0113 (13)
C67	0.0456 (18)	0.041 (2)	0.0348 (18)	−0.0060 (15)	0.0022 (14)	−0.0212 (16)
C1	0.0270 (15)	0.0319 (19)	0.044 (2)	−0.0007 (13)	0.0069 (14)	−0.0022 (16)
C2	0.0298 (17)	0.040 (2)	0.056 (2)	0.0020 (15)	0.0137 (16)	−0.0059 (18)
C3	0.0318 (17)	0.032 (2)	0.056 (2)	−0.0013 (14)	0.0117 (16)	0.0075 (17)
C4	0.0314 (16)	0.0288 (18)	0.0372 (18)	−0.0050 (14)	0.0032 (13)	−0.0004 (15)
C5	0.0263 (15)	0.0321 (18)	0.0421 (19)	−0.0075 (13)	0.0061 (13)	−0.0132 (15)
C6	0.0290 (16)	0.043 (2)	0.054 (2)	−0.0148 (15)	0.0125 (15)	−0.0121 (18)
C7	0.0269 (15)	0.0275 (18)	0.051 (2)	−0.0034 (13)	0.0056 (14)	−0.0123 (16)
C8	0.0278 (16)	0.0316 (19)	0.055 (2)	−0.0055 (14)	0.0107 (15)	−0.0153 (17)
N2	0.055 (2)	0.069 (3)	0.074 (3)	−0.0022 (17)	0.0231 (18)	−0.032 (2)
N3	0.0396 (17)	0.061 (2)	0.068 (2)	0.0018 (15)	0.0005 (16)	0.0144 (19)
N4	0.0468 (17)	0.0364 (18)	0.0525 (19)	0.0050 (14)	0.0044 (14)	−0.0071 (15)
N6	0.059 (2)	0.088 (3)	0.055 (2)	−0.033 (2)	0.0127 (17)	−0.009 (2)
N7	0.0428 (16)	0.0360 (18)	0.066 (2)	−0.0129 (14)	0.0066 (14)	−0.0097 (16)
N8	0.0401 (16)	0.052 (2)	0.070 (2)	−0.0119 (14)	0.0044 (15)	−0.0304 (18)

### Geometric parameters (Å, º)

**Table:**

Fe1—N20	1.957 (2)	C36—C37	1.508 (4)
Fe1—N10	1.959 (2)	C37—H37A	0.9800
Fe1—N60	1.963 (2)	C37—H37B	0.9800
Fe1—N30	1.965 (2)	C37—H37C	0.9800
Fe1—N50	1.967 (2)	C41—C45	1.411 (4)
Fe1—N40	1.968 (2)	C42—C43	1.399 (4)
N10—C12	1.334 (3)	C42—H42	0.9500
N10—C11	1.372 (3)	C43—C44	1.361 (4)
N20—C22	1.339 (3)	C43—H43	0.9500
N20—C21	1.368 (3)	C44—C45	1.406 (4)
N30—C32	1.339 (3)	C44—H44	0.9500
N30—C31	1.365 (3)	C45—C46	1.443 (4)
N40—C42	1.331 (3)	C46—C47	1.510 (4)
N40—C41	1.359 (3)	C47—H47A	0.9800
N50—C52	1.335 (3)	C47—H47B	0.9800
N50—C51	1.365 (3)	C47—H47C	0.9800
N60—C62	1.337 (3)	C51—C55	1.404 (4)
N60—C61	1.369 (3)	C51—C61	1.420 (4)
C11—C15	1.394 (4)	C52—C53	1.383 (4)
C11—C21	1.423 (3)	C52—H52	0.9500
C12—C13	1.393 (4)	C53—C54	1.369 (4)
C12—H12	0.9500	C53—H53	0.9500
C13—C14	1.366 (4)	C54—C55	1.408 (4)
C13—H13	0.9500	C54—H54	0.9500
C14—C15	1.406 (4)	C55—C56	1.450 (4)
C14—H14	0.9500	C56—C66	1.369 (4)
C15—C16	1.454 (4)	C56—C57	1.511 (4)
C16—C26	1.371 (4)	C57—H57A	0.9800
C16—C17	1.507 (4)	C57—H57B	0.9800
C17—H17A	0.9800	C57—H57C	0.9800
C17—H17B	0.9800	C61—C65	1.399 (4)
C17—H17C	0.9800	C62—C63	1.394 (4)
C21—C25	1.394 (4)	C62—H62	0.9500
C22—C23	1.392 (4)	C63—C64	1.367 (4)
C22—H22	0.9500	C63—H63	0.9500
C23—C24	1.360 (4)	C64—C65	1.413 (4)
C23—H23	0.9500	C64—H64	0.9500
C24—C25	1.419 (4)	C65—C66	1.449 (4)
C24—H24	0.9500	C66—C67	1.501 (4)
C25—C26	1.443 (4)	C67—H67A	0.9800
C26—C27	1.506 (4)	C67—H67B	0.9800
C27—H27A	0.9800	C67—H67C	0.9800
C27—H27B	0.9800	C1—C4	1.397 (4)
C27—H27C	0.9800	C1—C3	1.402 (5)
C31—C35	1.398 (4)	C1—C2	1.411 (5)
C31—C41	1.419 (4)	C2—N2	1.155 (5)
C32—C33	1.394 (4)	C3—N3	1.156 (4)
C32—H32	0.9500	C4—N4	1.155 (4)
C33—C34	1.363 (4)	C5—C7	1.398 (4)
C33—H33	0.9500	C5—C8	1.403 (5)
C34—C35	1.411 (4)	C5—C6	1.413 (5)
C34—H34	0.9500	C6—N6	1.151 (4)
C35—C36	1.453 (4)	C7—N7	1.157 (4)
C36—C46	1.367 (4)	C8—N8	1.157 (4)
			
N20—Fe1—N10	82.89 (9)	C34—C35—C36	124.8 (3)
N20—Fe1—N60	94.50 (9)	C46—C36—C35	120.5 (3)
N10—Fe1—N60	89.17 (9)	C46—C36—C37	123.2 (3)
N20—Fe1—N30	89.92 (9)	C35—C36—C37	116.3 (3)
N10—Fe1—N30	95.52 (9)	C36—C37—H37A	109.5
N60—Fe1—N30	173.92 (9)	C36—C37—H37B	109.5
N20—Fe1—N50	176.49 (9)	H37A—C37—H37B	109.5
N10—Fe1—N50	94.93 (9)	C36—C37—H37C	109.5
N60—Fe1—N50	82.70 (9)	H37A—C37—H37C	109.5
N30—Fe1—N50	93.03 (9)	H37B—C37—H37C	109.5
N20—Fe1—N40	94.31 (9)	N40—C41—C45	124.1 (2)
N10—Fe1—N40	176.56 (9)	N40—C41—C31	115.8 (2)
N60—Fe1—N40	93.05 (9)	C45—C41—C31	120.1 (2)
N30—Fe1—N40	82.45 (9)	N40—C42—C43	121.7 (3)
N50—Fe1—N40	87.97 (9)	N40—C42—H42	119.1
C12—N10—C11	117.2 (2)	C43—C42—H42	119.1
C12—N10—Fe1	129.55 (19)	C44—C43—C42	120.6 (3)
C11—N10—Fe1	113.15 (15)	C44—C43—H43	119.7
C22—N20—C21	117.6 (2)	C42—C43—H43	119.7
C22—N20—Fe1	129.00 (19)	C43—C44—C45	119.8 (3)
C21—N20—Fe1	113.43 (16)	C43—C44—H44	120.1
C32—N30—C31	117.4 (2)	C45—C44—H44	120.1
C32—N30—Fe1	129.23 (19)	C44—C45—C41	115.9 (2)
C31—N30—Fe1	113.32 (17)	C44—C45—C46	124.7 (3)
C42—N40—C41	117.8 (2)	C41—C45—C46	119.3 (3)
C42—N40—Fe1	129.02 (18)	C36—C46—C45	120.4 (3)
C41—N40—Fe1	113.08 (17)	C36—C46—C47	122.3 (3)
C52—N50—C51	117.3 (2)	C45—C46—C47	117.4 (3)
C52—N50—Fe1	129.58 (19)	C46—C47—H47A	109.5
C51—N50—Fe1	112.98 (17)	C46—C47—H47B	109.5
C62—N60—C61	117.4 (2)	H47A—C47—H47B	109.5
C62—N60—Fe1	129.68 (19)	C46—C47—H47C	109.5
C61—N60—Fe1	112.91 (17)	H47A—C47—H47C	109.5
N10—C11—C15	124.3 (2)	H47B—C47—H47C	109.5
N10—C11—C21	115.3 (2)	N50—C51—C55	124.1 (2)
C15—C11—C21	120.5 (2)	N50—C51—C61	115.4 (2)
N10—C12—C13	122.2 (3)	C55—C51—C61	120.5 (2)
N10—C12—H12	118.9	N50—C52—C53	122.5 (3)
C13—C12—H12	118.9	N50—C52—H52	118.7
C14—C13—C12	120.2 (2)	C53—C52—H52	118.7
C14—C13—H13	119.9	C54—C53—C52	120.3 (3)
C12—C13—H13	119.9	C54—C53—H53	119.8
C13—C14—C15	119.9 (2)	C52—C53—H53	119.8
C13—C14—H14	120.1	C53—C54—C55	119.7 (3)
C15—C14—H14	120.1	C53—C54—H54	120.2
C11—C15—C14	116.3 (3)	C55—C54—H54	120.2
C11—C15—C16	119.0 (2)	C51—C55—C54	116.1 (3)
C14—C15—C16	124.8 (2)	C51—C55—C56	119.0 (2)
C26—C16—C15	120.5 (2)	C54—C55—C56	124.9 (3)
C26—C16—C17	122.1 (3)	C66—C56—C55	120.5 (3)
C15—C16—C17	117.4 (2)	C66—C56—C57	122.9 (3)
C16—C17—H17A	109.5	C55—C56—C57	116.7 (3)
C16—C17—H17B	109.5	C56—C57—H57A	109.5
H17A—C17—H17B	109.5	C56—C57—H57B	109.5
C16—C17—H17C	109.5	H57A—C57—H57B	109.5
H17A—C17—H17C	109.5	C56—C57—H57C	109.5
H17B—C17—H17C	109.5	H57A—C57—H57C	109.5
N20—C21—C25	124.5 (2)	H57B—C57—H57C	109.5
N20—C21—C11	115.2 (2)	N60—C61—C65	124.4 (2)
C25—C21—C11	120.3 (2)	N60—C61—C51	115.6 (2)
N20—C22—C23	121.8 (3)	C65—C61—C51	120.1 (2)
N20—C22—H22	119.1	N60—C62—C63	122.0 (3)
C23—C22—H22	119.1	N60—C62—H62	119.0
C24—C23—C22	120.5 (2)	C63—C62—H62	119.0
C24—C23—H23	119.8	C64—C63—C62	120.4 (3)
C22—C23—H23	119.8	C64—C63—H63	119.8
C23—C24—C25	120.0 (3)	C62—C63—H63	119.8
C23—C24—H24	120.0	C63—C64—C65	119.7 (3)
C25—C24—H24	120.0	C63—C64—H64	120.1
C21—C25—C24	115.7 (2)	C65—C64—H64	120.1
C21—C25—C26	119.6 (2)	C61—C65—C64	116.1 (3)
C24—C25—C26	124.7 (3)	C61—C65—C66	119.5 (2)
C16—C26—C25	120.2 (3)	C64—C65—C66	124.4 (3)
C16—C26—C27	122.8 (3)	C56—C66—C65	120.3 (3)
C25—C26—C27	117.1 (2)	C56—C66—C67	123.4 (3)
C26—C27—H27A	109.5	C65—C66—C67	116.3 (3)
C26—C27—H27B	109.5	C66—C67—H67A	109.5
H27A—C27—H27B	109.5	C66—C67—H67B	109.5
C26—C27—H27C	109.5	H67A—C67—H67B	109.5
H27A—C27—H27C	109.5	C66—C67—H67C	109.5
H27B—C27—H27C	109.5	H67A—C67—H67C	109.5
N30—C31—C35	124.3 (3)	H67B—C67—H67C	109.5
N30—C31—C41	115.2 (2)	C4—C1—C3	119.6 (3)
C35—C31—C41	120.5 (2)	C4—C1—C2	119.9 (3)
N30—C32—C33	121.9 (3)	C3—C1—C2	120.4 (3)
N30—C32—H32	119.0	N2—C2—C1	179.3 (4)
C33—C32—H32	119.0	N3—C3—C1	178.1 (4)
C34—C33—C32	120.4 (3)	N4—C4—C1	179.2 (4)
C34—C33—H33	119.8	C7—C5—C8	120.0 (3)
C32—C33—H33	119.8	C7—C5—C6	119.8 (3)
C33—C34—C35	119.8 (3)	C8—C5—C6	120.2 (3)
C33—C34—H34	120.1	N6—C6—C5	179.5 (4)
C35—C34—H34	120.1	N7—C7—C5	178.3 (4)
C31—C35—C34	116.1 (3)	N8—C8—C5	179.3 (4)
C31—C35—C36	119.1 (3)		

### Hydrogen-bond geometry (Å, º)

**Table:**

*D*—H···*A*	*D*—H	H···*A*	*D*···*A*	*D*—H···*A*
C52—H52···N30	0.95	2.59	3.070 (3)	112
C22—H22···N40	0.95	2.59	3.089 (4)	113
C32—H32···N8	0.95	2.43	3.266 (4)	146
C42—H42···N60	0.95	2.57	3.056 (3)	112
C22—H22···N6^i^	0.95	2.54	3.271 (4)	134
C12—H12···N2^ii^	0.95	2.55	3.352 (4)	142
C62—H62···N3^iii^	0.95	2.51	3.266 (4)	137
C44—H44···N7^iv^	0.95	2.59	3.312 (4)	133

Symmetry codes: (i) −*x*+1, −*y*+1, −*z*+1; (ii) *x*+1, *y*, *z*; (iii) −*x*, −*y*+1, −*z*; (iv) *x*−1, *y*−1, *z*.
